# Supplementary material for: Bilinguals are better than monolinguals in detecting manipulative discourse
Source: PLoS One. 2021 Sep 7;16(9):e0256173. doi: 10.1371/journal.pone.0256173 (PMC8423309; doi:10.1371/journal.pone.0256173)
Supplement: S1 File — 1a. Probability of giving a ‘neither correct nor wrong’ response by condition and group. 1b. ‘Neither’ responses: post-hoc pairwise comparisons. 2a. Accuracy as predicted by an interaction of condition and group. 2b. Accuracy: post-hoc pairwise comparisons. 3a. Reaction times (fillers and illusions together) as predicted by an interaction of judgement type and group, with age as a main effect. 3b. Reaction times (fillers and illusions together): post-hoc pairwise comparisons. 4a. Reaction times (illusions) as predicted by an interaction of judgement type and group, age as a main effect. 4b. Reaction times (illusions): post-hoc pairwise comparisons. 5a. Reaction times (fillers and illusions together, ‘neither’ responses excluded) as predicted by an interaction of judgement type, condition and group, with age as a main effect. 5b. Reaction times (fillers and illusions together, ‘neither’ responses excluded): post-hoc pairwise comparisons. 6a. Accuracy as predicted by an interaction of condition and length of bilingual experience group. 6b. Accuracy: post-hoc pairwise comparisons. 7a. Reaction times (fillers and illusions together) as predicted by as an interaction of condition and length of bilingual experience group, with age as a main effect. 7b. Reaction times: post-hoc pairwise comparisons. 8a. Reaction times (illusions) as predicted by an interaction of condition and length of bilingual experience group, with age as a main effect. 8b. Reaction times (illusions): post-hoc pairwise comparisons. (DOCX) [file pone.0256173.s001.docx]

**Bilinguals are better than monolinguals in detecting manipulative discourse**

Evelina Leivada,^1^ Natalia Mitrofanova,^2^ Marit Westergaard^2,3^

^1^ Universitat Rovira i Virgili

^2^ UiT-The Arctic University of Norway

^3^ NTNU Norwegian University of Science and Technology

**Supplementary Materials**

Details of the statistical analysis

S1a. Probability of giving a ‘neither correct nor wrong’ response by condition and group

1b. ‘Neither’ responses: post-hoc pairwise comparisons

2a. Accuracy as predicted by an interaction of condition and group

2b. Accuracy: post-hoc pairwise comparisons

3a. Reaction times (fillers and illusions together) as predicted by an interaction of judgement type and group, with age as a main effect

3b. Reaction times (fillers and illusions together): post-hoc pairwise comparisons

4a. Reaction times (illusions) as predicted by an interaction of judgement type and group, age as a main effect

4b. Reaction times (illusions): post-hoc pairwise comparisons

5a. Reaction times (fillers and illusions together, ‘neither’ responses excluded) as predicted by an interaction of judgement type, condition and group, with age as a main effect

5b. Reaction times (fillers and illusions together, ‘neither’ responses excluded): post-hoc pairwise comparisons

6a. Accuracy as predicted by an interaction of condition and length of bilingual experience group

6b. Accuracy: post-hoc pairwise comparisons

7a. Reaction times (fillers and illusions together) as predicted by as an interaction of condition and length of bilingual experience group, with age as a main effect

7b. Reaction times: post-hoc pairwise comparisons

8a. Reaction times (illusions) as predicted by an interaction of condition and length of bilingual experience group, with age as a main effect

8b. Reaction times (illusions): post-hoc pairwise comparisons
