# Supplementary material for: Bilinguals are better than monolinguals in detecting manipulative discourse
Source: PLoS One. 2021 Sep 7;16(9):e0256173. doi: 10.1371/journal.pone.0256173 (PMC8423309; doi:10.1371/journal.pone.0256173)
Supplement: Attachment — Submitted filename: Review PLOS ONE.docx [file pone.0256173.s002.docx]

Review for PLOS ONE

Title: “Bilinguals are better than monolinguals in detecting manipulative discourse”

Reviewer: Anna Pineda

The paper is original in as much it looks into bilingualism effects on the domain of linguistic illusions, a type of linguistic stimulus that had not been tested from this perspective so far. The authors frame the necessity and advantages of this research within the line of studies on the so-called *the bilingual advantage*. Put very simply, they explain that if bilinguals are supposed to have a better cognitive aptitude (speed of computation, mental flexibility, attention orienting...), then this could correlate with a better performance in tasks which aim to trick the cognitive parser. According to them, studying this kind of contexts especially when it comes to *linguistic tricks* or *illusions* is clearly relevant. This reveals to be a promising area of study with respect to the finding of robust effects of bilingualism to cognition.

In general terms, the experiment is clearly contextualized, the results are presented in an appropriate way, and the discussion of the results is satisfactory, including the interesting hypothesis which is presented, which nuances the expectations of the so-called *the bilingual advantage* and argues for a view of bilingualism and plurilingualism as nuanced spectrum experiences rather as dichotomous categories.

I enjoyed reading the paper and I am happy to recommend it for publication. I have some comments, most of them minor ones. I explain them below, following the order of the paper.

- When Müller-Lye illusion is exemplified on pg. 3, I think real arrows should be used, because building an arrow with Word symbols does not produce the illusion they talk about, since there are blank spaces between the central line and the endings of the arrow –if anything, their illustrations produce the opposite illusion they want to show! I did not know about this illusion and had to search it in order to see an adequate illustration of it. Actually, after having written this, I see that the authors themselves say exactly the same thing: “If the context is altered such that the arrows are presented separately before the line [REVIEWER: which is what the authors do in the paper!], the Müller-Lyer illusion disappears and the effect that emerges goes in the opposite direction (i.e., the line that temporally succeeds the inward arrows is found to be shorter)”
- It is only on pg. 5 that we are informed that the study focuses on Greek bilinguals. I think mentioning this way before would be better, since until then only English examples are provided and one assumes that the experiment targets this language. Actually, even if they want to keep providing the information only on page 5, they should reverse how they do it, because they first say, in passing, that “this effect of repeatability does not exist in Greek” (so the reader must infer that Greek is the language they test) and it is not only until the next paragraph where we find the claim that we would have wanted to know from the beginning: “The language of testing was Greek.”
- Another crucial thing would be to provide in the text some examples of the Greek sentences that were used in the test. I imagine they can all be found in the “Supplementary files” folder, which I was not able to downlead, but in any case, I would really have appreciated some examples in the main text, as only English examples are provided, while this is not the language targeted.
- I see that all the bilinguals were speakers of Greek and a Germanic language. On the one side, I wonder whether the same results would have been obtained if the other language were a non-Indo-European one. I don’t know if there exists literature on this topic. On the other side, I think the paper would benefit from mentioning other studies focusing on the particular case of Germanic-Greek bilingualism.
- Regarding the claim “Τhe lower limit of the period of residence outside Greece was set to 4 years (average: 11.9, SD: 9.9), in order to establish prolonged periods of L1 inhibition and exposure to an L2/3”, I wonder whether there is any reference the authors can mention to support the choice of this limit (4 years). Likewise, the authors could mention some previous research on how a more or less prolonged period of L1 inhibition / exposure to an L2/L3 influences the bilinguals performance in this sort of tasks, in order to support and contextualize the choice they make, even if it may seem *obvious* from their expert point of view.
- The authors mention that among exclusion criteria there was “inappropriate behavior (i.e., a series of non-target answers) with the fillers in the task”, but could they say how many cases like these did they find, if any?
- When the authors say: “The results showed a better performance of the bidialectal group in terms of rejecting the illusions as ill-formed, but this finding could not be unambiguously attributed to an effect of linguistic background, as several other factors might also play a role [29]”, I understand they refer the readers to this other work, but I think one would appreciate if they could provide some examples of these “other factors”.
- On page 7, the authors mention that “Given that older participants might reasonably take longer to respond, age was added as a main effect.” However, they don’t provide any specific information on this, while the readers are expecting it (or at least I was). It is not until page 9, after having addressed many other issues, that they explain “As mentioned already, age is an expected covariate with length of language use. However, the results did not associate the oldest subgroup with longer reaction times” and provide the corresponding table (where age is related to the length of the bilingual experience). I think this ordering is confusing. They can keep this ordering but on page 7, when they mention the *age* variable for the first time, I think they should refer to the part of the study where they are going to deal with it.
- The authors say “Participants who spent more than 7 years in a context that required L1 suppression formed the “long bilingual experience” group (68 participants, average age: 44.2). Participants with 7 or less years of bilingual experience were grouped into the “short bilingual experience” group (70 participants, average age: 33.1). Monolinguals with no or scarce experience of L1 suppression counted as a separate group (138 participants).” However, I am not sure that spending more than 7 years abroad always automatically entails a greater L1 suppression: that is, someone may live abroad for 10 years but continue to speak Greek with their family (wife/husband & kids, for example), who moved with them vs. someone who has been living abroad only 3 years but with no friends or family to talk to on a daily basis. Could the authors clarify whether they took into account all these variables when dividing the bilinguals into two groups and, if not (as it seems to become obvious in the final sentences of the paper), could they explain how did they establish such a correlation?
- A final observation I would like to make, which is maybe due to my lack of familiarity with this type of studies, has to do with the fact that the authors talk about “the question of whether bilingualism can decrease susceptibility to manipulative discourse”. They then analyze a series of grammatical illusions, but I was wondering whether their mentions of *manipulative discourse* were supposed to imply something else beyond that sort of illusions. It may be the case that *manipulative discourse* it’s used here in a restricted, technical sense, but at first I was expecting some kind of generalization or transposition of the findings beyond the confinement of comparative sentences, etc. especially because these words are included in the title and are normally by used by our societies in a much broader sense.
