# Supplementary material for: Bilinguals are better than monolinguals in detecting manipulative discourse
Source: PLoS One. 2021 Sep 7;16(9):e0256173. doi: 10.1371/journal.pone.0256173 (PMC8423309; doi:10.1371/journal.pone.0256173)
Supplement: Attachment — Submitted filename: Response Letter.docx [file pone.0256173.s003.docx]

***Response Letter***

We would like to express our gratitude to the editor and the two reviewers whose comments proved very useful in developing our arguments better. In this letter, we address all the points raised by the reviewers and link them to the related changes in the revised manuscript.

**Reviewer’s #1 comments**

*1. It should be better described how the task employed measures the language control and processing at the same time. In the experiment, participants are asked to indicate if a given sentence is grammatically correct or not. So, the correct response for the illusions is “wrong”. Thus, accuracy differences between groups then indicate that bilinguals were more accurate indicating that a sentence was ungrammatical despite being acceptable. Are those results indicative that acceptability is a more automatic judgement and it needs to be suppressed in order to answer to the grammatically judgement?*

The instruction given to the participants was to judge whether the sentences they were presented with sounded well-formed to them. In this sense, it was a not a binary yes(grammatical)/ no(ungrammatical) choice, but an acceptability judgment of well-formedness based on 3-point Likert scale. Although we agree with the reviewer that this is a very interesting topic, we do not think our experiment provided results that speak about the distinction of grammaticality and acceptability in terms of whether one is more automatic than the other or which one comes first. The reason for this is that we only asked for one type of judgment. We did not ask participants whether they thought that the tested sentences violated one or more rules of grammar. From a conceptual point of view, if we were to answer the last question the reviewer raises in the above comment, the answer could well be positive. However, precisely because we do not have results to support this answer, we feel unable to substantially comment on this in a way that benefits the manuscript. Of course, we are very grateful to the reviewer for pointing out this important aspect and in future work we will attempt to broach this issue.

*The results are interpreted in the realm of a trade-off accuracy, but applying to the bilingual experience rather than to the task itself. Could not be the case that this task is a two-step processing entailing a suppression of the automatic response followed by a grammaticality judgement? And that only those participants that accurately suppress the acceptability judgement (the sentence is grammatically acceptable), have a second step, slower, where the grammatical judgement has to be made?*

We agree with the reviewer. In the revised version, we make explicit reference to the fact that this is a two-step process on p. 4.

*The method section is too convoluted making very difficult to know what the exact design was. Information regarding the participants, materials and design is mixed. Subsections of methods including participants, design, procedure and data analysis would help to have this information organized.*

The reviewer is right. In the revised version, the methods section has been split to subsections and the information about participants, design, and procedure is no longer mixed.

*Regarding the design (page 5, end of second paragraph), as it is written now, it is very difficult to follow. There were 10 experimental sentences, all of them ungrammatical. So, the sentence “15 ungrammatical and 5 grammatical” only applies to the fillers. Is this correct? Likewise, it says: ”This means that, excluding fillers, 5520 data points sere collected, 2760 for each measure”. Which measure? If fillers are excluded and there were 10 illusions, this leads to 2760 data points.*

The phrasing was unclear. We have now rephrased this part (and we reworked and reorganized the methods section in general). With respect to this specific point, it is 2760 datapoints per measure: off-line (acceptability judgments) and on-line (reaction times), thus 5520 in total.

*My major concern with these data is that conclusions are based on performance for 10 sentences; How stable is the data regarding these sentences?*

Yes, there are 10 test items that feature illusions. Indeed, it is a small number of stimuli, but within the limits of what is standardly accepted in experimental syntax. To give some concrete references for comparisons, tutorials for experimental syntax advocate a minimum of 3 items per condition: <http://www.ciscl.unisi.it/doc/doc_ev/Schippers%202016%20Tutorial_Experimental_Syntax.pdf>

Also, one of the landmark references for comparative illusions, Wellwood et al. 2018 in the *Journal of Semantics*, had split test items in different lists, such that every participant saw only 6 items from the Illusion Condition and 18 items from the Filler Condition. In comparison, we had 10 items that involve illusions and 20 fillers for each participant.

*It would be interesting to see the plot including datapoints for participants or items to see behavior at the individual level.*

Please see below the by-participant plots. We did not include them in the manuscript because we think they are not as informative as the plots we have already provided, but if the reviewer and/or the editor would like us to add them, we would be happy to.

Here are by-item plots for the two conditions:

*It is unclear why the age factor was included in the analysis. Especially for the comparison of bilinguals and monolinguals, where the two groups did not differ in terms of age. How the model converges without the age factor? Why age and not education? A first analysis should include these factors and test their influence on the accuracy in detecting illusions.*

The age factor was included because the literature suggests that older people are slower in cognitive tasks that involve reaction times than younger people, and our task includes a time component. To the best of our knowledge, education has not been claimed to have an impact on reaction times. Also, our two groups, monolinguals and bilinguals, are matched for level of education.

*Surprisingly, the age factor is not included in the three group comparison, where age differences seem more obvious. A statistical test should indicate if the 3 groups differed in age.*

In the revised manuscript, the information regarding age in the three group comparison is given on p. 10: “To analyze the results statistically, we fit a linear model predicting the common log reaction time as an interaction of Condition and Length of bilingual experience group, with age added as a separate main effect” and “As mentioned already, age is an expected covariate with length of language use. However, the results did not associate the oldest subgroup with longer reaction times. The group of bilinguals with long bilingual experience is significantly older than the group with short bilingual experience (Levene’s Test for equality of variance F=10.997, p=0.001), but, as figure 7 shows, they responded faster than the younger bilingual group, in both illusions and fillers.”

*Grammatically judgements are made in Greek, which for many participants living abroad, Greek must become their second language. It should be clarified if differences between bilinguals and monolinguals show the bilingual use of two languages (relative to only one of monolinguals) or processing in L1 in monolinguals vs L2 in bilinguals*.

All participants stated that Greek was their L1. Moreover, they were all recruited online, through advertisements in the respective Greek communities in their countries of residence, something that suggests that they all have contact with Greek on a daily basis. We thus think that differences emerge from the use of two or more languages in the bilingual group, and more specifically, from the suppression of L1 and the consequences this entails.

*Related to this, page 8 last paragraph starts with “zooming in on the performance of the bilingual group, we observed that the length of bilingual experience correlates with the ability to detect illusions”. No correlation analysis is provided.*

We reformulated the paragraph stating that the length of bilingual exposure *was a significant predictor* of accuracy. Participants with short bilingual experience (7 years or less) were significantly better than the monolinguals and the participants with longer bilingual experience (above 7 years). Participants with longer bilingual experience were significantly better than the monolinguals, but worse than participants with shorter bilingual experience.

*Is there a reason for splitting bilinguals in two groups instead of considering experience as a continuous variable?*

The relation of the length of bilingual experience with accuracy was non-linear (significantly higher accuracy in the group with shorter bilingual experience, and a gradual decrease in the group with longer bilingual experience). If we take the length of bilingual exposure as a continuous variable, we’d have a big discrepancy in terms of data points for individual values of the variable: data from 138 participants for 0 years of L2 exposure (monolinguals), but much less data for each subsequent year: e.g., 21 participants with 4 years of exposure, 27 participants with 5 years of exposure, 1 participant for 8 years of exposure, etc.; please see the histogram below).

Minor points

A table with means and statistics for the two/three groups should be included.

Two tables have been added on pp. 8 and 10.

*More information should be added regarding the language experience of the three groups.*

More information has been added in the second paragraph of the participant section. If the reviewer thinks that specific information is missing, we will be happy to add it.

*It is not mentioned in the text, but I guess reaction times only included correct responses.*

We have presented and made publicly available the entire dataset. On p. 8 of the revised manuscript, reaction times are reported separately per Judgment type, including a separate report for the correct responses. The reaction times of the correct responses are also presented in figures 5 and 6 of the revised manuscript for illusions and fillers respectively.

*There are some typos in the text. For instance, page 4 end second paragraph, to address different monolinguals interlocutors, should be monolingual interlocutors.*

It has been corrected.

*Discussion first paragraph. Bilinguals are better than. It would be more appropriate bilinguals are more accurate than.*

It has been corrected. We thank the reviewer once again for the very helpful comments.

**Reviewer’s #2 comments**

*When Müller-Lye illusion is exemplified on pg. 3, I think real arrows should be used, because building an arrow with Word symbols does not produce the illusion they talk about, since there are blank spaces between the central line and the endings of the arrow –if anything, their illustrations produce the opposite illusion they want to show! I did not know about this illusion and had to search it in order to see an adequate illustration of it. Actually, after having written this, I see that the authors themselves say exactly the same thing: “If the context is altered such that the arrows are presented separately before the line [REVIEWER: which is what the authors do in the paper!], the Müller-Lyer illusion disappears and the effect that emerges goes in the opposite direction (i.e., the line that temporally succeeds the inward arrows is found to be shorter)”*

The reviewer is right in pointing out this important omission. In the revised manuscript, a new figure 1 has been added on p. 3.

*It is only on pg. 5 that we are informed that the study focuses on Greek bilinguals. I think mentioning this way before would be better, since until then only English examples are provided and one assumes that the experiment targets this language. Actually, even if they want to keep providing the information only on page 5, they should reverse how they do it, because they first say, in passing, that “this effect of repeatability does not exist in Greek” (so the reader must infer that Greek is the language they test) and it is not only until the next paragraph where we find the claim that we would have wanted to know from the beginning: “The language of testing was Greek.”*

In the revised manuscript, the section ‘Method’ has been reworked, restructured, and split in subsections. The order of the relevant parts that the reviewer mentions in the above comment has been reversed as the reviewer suggested.

*Another crucial thing would be to provide in the text some examples of the Greek sentences that were used in the test. I imagine they can all be found in the “Supplementary files” folder, which I was not able to downlead, but in any case, I would really have appreciated some examples in the main text, as only English examples are provided, while this is not the language targeted.*

Apologies for this omission. In the revised version, we have added examples on p. 6.

*I see that all the bilinguals were speakers of Greek and a Germanic language. On the one side, I wonder whether the same results would have been obtained if the other language were a non-Indo-European one. I don’t know if there exists literature on this topic. On the other side, I think the paper would benefit from mentioning other studies focusing on the particular case of Germanic-Greek bilingualism.*

We appreciate this comment as this is a matter to which we have also devoted a lot of thought. The topic of illusions has been approached through testing monolingual speakers of English. There are also two studies with speakers of Danish, plus anecdotal evidence that the illusion is robust in many other languages. Given that the controlled experiments (all of which are mentioned in our study) involve only monolingual speakers, there is no literature that can help us answer the very sensible and indeed very interesting question that the reviewer raises. If we were to provide an answer on purely speculative grounds, we think that the results may not have been the same if a non-Indo-European language was added in the mix. In the future, the plan is to adapt this experiment to bilingual speakers of Spanish and Basque in order to verify this hypothesis. However, at present we do not have solid arguments or results that can confirm or disconfirm this speculation, therefore we decided to refrain from commenting on this in the manuscript and focus instead on the tested population.

With respect to mentioning other studies on Greek-Germanic bilingualism, although there are a few, to the best of our knowledge, they do not bear a strong resemblance or relevance to our results, because they study specific grammatical phenomena and the way they are attested in the target language(s).

*Regarding the claim “Τhe lower limit of the period of residence outside Greece was set to 4 years (average: 11.9, SD: 9.9), in order to establish prolonged periods of L1 inhibition and exposure to an L2/3”, I wonder whether there is any reference the authors can mention to support the choice of this limit (4 years). Likewise, the authors could mention some previous research on how a more or less prolonged period of L1 inhibition / exposure to an L2/L3 influences the bilinguals performance in this sort of tasks, in order to support and contextualize the choice they make, even if it may seem obvious from their expert point of view.*

As there is no literature on the topic, we made a call to set the limit to 4 years based on indirect evidence. Many studies assume that attrition starts after 5-6 years of residence at the L2 community (e.g., Kaltsa et al. 2015 in Lingua). However, these studies are interested in attrition and aspects of potential influence of L2 to L1 (i.e. how specific grammatical characteristics of L2 affect the equivalent grammatical characteristics in L1) and vice versa. For such studies, it is indeed very important to establish the presence of attrition and its starting point. Our experiment, however, does not tap into this, and in fact, it is unlikely (probably impossible) that our bilinguals were consistently exposed to linguistic illusions in their L2 community such that their perception/judgment of illusions in their L1 would be affected by the grammar of their L2. We thus didn´t adopt the attrition standard for a minimum stay in the L2 community (if such a standard exists at all; different researchers propose different minimums), but we did want to have a prolonged period of stay in the L2 community. We thus decided to set the limit to 4 years (right before attrition starts) as the absolute minimum that seemed reasonable to us in the context of this exploratory research.

*The authors mention that among exclusion criteria there was “inappropriate behavior (i.e., a series of non-target answers) with the fillers in the task”, but could they say how many cases like these did they find, if any?*

This information has now been added on p. 6.

*When the authors say: “The results showed a better performance of the bidialectal group in terms of rejecting the illusions as ill-formed, but this finding could not be unambiguously attributed to an effect of linguistic background, as several other factors might also play a role [29]”, I understand they refer the readers to this other work, but I think one would appreciate if they could provide some examples of these “other factors”.*

This information has now been added on p. 7.

*On page 7, the authors mention that “Given that older participants might reasonably take longer to respond, age was added as a main effect.” However, they don’t provide any specific information on this, while the readers are expecting it (or at least I was). It is not until page 9, after having addressed many other issues, that they explain “As mentioned already, age is an expected covariate with length of language use. However, the results did not associate the oldest subgroup with longer reaction times” and provide the corresponding table (where age is related to the length of the bilingual experience). I think this ordering is confusing. They can keep this ordering but on page 7, when they mention the age variable for the first time, I think they should refer to the part of the study where they are going to deal with it.*

The order has been changed. In the revised manuscript we explain why we test the age variable on p. 7.

*The authors say “Participants who spent more than 7 years in a context that required L1 suppression formed the “long bilingual experience” group (68 participants, average age: 44.2). Participants with 7 or less years of bilingual experience were grouped into the “short bilingual experience” group (70 participants, average age: 33.1). Monolinguals with no or scarce experience of L1 suppression counted as a separate group (138 participants).” However, I am not sure that spending more than 7 years abroad always automatically entails a greater L1 suppression: that is, someone may live abroad for 10 years but continue to speak Greek with their family (wife/husband & kids, for example), who moved with them vs. someone who has been living abroad only 3 years but with no friends or family to talk to on a daily basis. Could the authors clarify whether they took into account all these variables when dividing the bilinguals into two groups and, if not (as it seems to become obvious in the final sentences of the paper), could they explain how did they establish such a correlation?*

The reviewer is right. It is not always the case that a longer stay in the L2 community entails a greater L1 suppression. We did not use targeted measures to quantify this, however we do have a fair idea of the demographic profile of the bilingual participants. They were all recruited through advertisements in the Greek communities in their countries of residence and they all have contact with Greek on a daily basis. Also it is likely that even people who relocated abroad alone have some contact with their Greek-speaking relatives or friends through technological means. We thus decided to take the length of bilingual experience into account, following the literature that suggests that the use of two languages progressively becomes a more automated switching process as one moves farther away from the onset of bilingualism (e.g., Paap 2018, mentioned on p. 12 in the revised manuscript)

*A final observation I would like to make, which is maybe due to my lack of familiarity with this type of studies, has to do with the fact that the authors talk about “the question of whether bilingualism can decrease susceptibility to manipulative discourse”. They then analyze a series of grammatical illusions, but I was wondering whether their mentions of manipulative discourse were supposed to imply something else beyond that sort of illusions. It may be the case that manipulative discourse it’s used here in a restricted, technical sense, but at first I was expecting some kind of generalization or transposition of the findings beyond the confinement of comparative sentences, etc. especially because these words are included in the title and are normally by used by our societies in a much broader sense.*

We understand what the reviewer says and we agree. It could be the case that the results we obtained extend to manipulative discourse more broadly, outside the context of grammatical illusions. Although we plan to test this in the future, at present we are not able to offer an answer about whether this is the case or not. However, we do realize that this point about the absence of a generalization is important and we have added it in the outlook, when addressing the limitations of this study.
